# Contract Incompleteness and the Boundaries of the Firm in Times of COVID-19

**DOI:** 10.1007/s10842-022-00387-7

**Published:** 2022-06-28

**Authors:** Marta Bernasconi, Sara Galetti, Valeria Gattai, Piergiovanna Natale

**Affiliations:** 1 Department of Economics, Management and Statistics, Università degli Studi di Milano-Bicocca, Piazza Ateneo Nuovo 1, 20126 Milan, Italy; 2 Center for European Studies (CefES), Università degli Studi di Milano-Bicocca, Piazza Ateneo Nuovo 1, 20126 Milan, Italy

**Keywords:** Contract incompleteness, Firm boundaries, COVID-19, Italy, Firm-level survey data, F23, D23, C35

## Abstract

In this paper, we study the firm’s boundaries in times of COVID-19. Ownership and location decisions govern sourcing and shape firms’ boundaries. Adopting incomplete contracts theory/international economics perspective, we investigate the determinants of ownership and location decisions and explore COVID-19–induced changes in firms’ boundaries. Drawing on survey data from a sample of Italian firms, our estimates suggest that input specificity drives the ownership decision, whereas the location decision depends on productivity. Few firms re-consider sourcing because of the pandemic, suggesting a great deal of inertia in firm’s boundaries.

## Introduction

Over the last few decades, the global economy has undergone two major changes that have reshaped production and trade. One pertains to the increasingly integrated nature of world markets, fuelled by trade liberalisation, regional integration agreements, falling transportation costs, and rapid technological advances (Baldwin and Venables 2013; Antras and de Gortari 2020). The second concerns the disintegration of production processes and the strategic dispersion of different value-added activities in ‘global value chains’ (Antras 2020; Kaplinsky 2020), also called ‘global supply chains’ (Baldwin 2012; Hernandez et al. 2014), or ‘global production sharing’ (Ng and Yeats 1999; Yeats 2001). These labels suggest that production processes embody value-added from multiple countries, with each country specialising in a particular production task rather than manufacturing final goods from conception to delivery. Firms increasingly participate in global value chains (GVCs), integrating backward as buyers of intermediate inputs or forward as suppliers of intermediate inputs, or both (Antras 2003; Antras and Chor 2013). The combination of world markets integration and production processes disintegration has reshaped the firm’s boundaries, producing various configurations in which some production tasks are internalised and others are externalised in the domestic country or abroad (Feenstra and Hanson 1996; Feenstra 1998).

Defined as the global economy’s backbone and central nervous system, GVCs were regarded as unstoppable (Kano et al. 2020) until very recently, when they slowed down—if not completely stopped—because of COVID-19. Javorcik (2020) argued that the pandemic posed an existential threat to GVCs. In the past, health-related crises and supply chain disruptions occurred because of SARS, Ebola, and the swine flu epidemic, with adverse effects on the global economy and trade. However, the socio-economic impact of COVID-19 is expected to be more severe, comparable with that of the Great Depression of 1929, the Second World War, and the Great Recession of 2008–2009 (Kowalski 2020). Participation in GVCs greatly increases countries’ and firms’ exposure to the epidemic shock as it entails that a disruption in any upstream stage ripples down, investing downward stages. Likewise, any adverse shock affecting downward stages transmits upwards, causing disruptions in markets that would otherwise be unaffected by the shock. The COVID-19 pandemic, causing upstream and downstream disruptions, poses new challenges for firms stretching their boundaries across countries (Baldwin et al. 2020). A shock of this magnitude is expected to create a discontinuous shift in the preferences and expectations of consumers, firms, and organisations. It is likely to produce significant and widespread effects in the short, medium, and long run.

Following Antras (2020), we believe that the impact of COVID-19 on GVCs can be fully assessed only at the firm level, investigating the firm’s boundaries. This is because participation in GVCs amounts to a joint ownership and location decision (Antras and Helpman 2004). For simplicity, consider a stylised framework in which the production of a final good requires intermediate inputs. In this context, the final good producer takes two crucial decisions over sourcing. The final good producer has to decide whether to make the components itself or buy them from an independent input supplier (ownership decision); conversely, it has to decide whether to employ domestic inputs or foreign inputs (location decision). In this framework, studying the firm’s boundaries means addressing sourcing issues at the crossroad between ownership and location considerations. Previously, sourcing was a local phenomenon; the firm’s boundaries could be fully explained in terms of the ownership decision, the choice being restricted to make-or-buy. Nowadays, sourcing has become a global phenomenon; therefore, the firm’s boundaries should be addressed considering ownership and location decisions jointly, the choice involving the make-or-buy, and the domestic-or-foreign alternatives.

In this paper, we empirically study the firm’s boundaries in times of COVID-19. For the present research, we provide new empirical evidence on a large stratified sample of Italian manufacturing firms headquartered in Lombardy, one of the most developed regions in Europe and, concurrently, one of the first regions severely affected by the COVID-19 pandemic. Particularly, our evidence draws on survey interviews conducted between April and July 2020 of a sample of 212 Italian firms headquartered in the region and stratified by size, manufacturing activity, and province.

Considering the incomplete contracts theory/international economics perspective, we study the sampled firms’ boundaries in times of COVID-19, discussing the extent to which input specificity and productivity affect ownership and location decisions. This allows us to portray sourcing strategies at the onset of the pandemic. Moreover, we investigate the sampled firm’s plans to permanently rethink their ownership and location decisions because of the pandemic. This allows us to capture the expected medium- and long-run effects of COVID-19 on sourcing.

Based on survey data, our sample’s ownership decision involves relying on independent input suppliers the most, engaging in outsourcing more than insourcing. Regarding location, Lombardy firms employ domestic inputs the most, engaging in domestic sourcing more than foreign sourcing.

Our descriptive statistics and econometric analysis suggest that the ownership decision is driven by input specificity, whereas the location decision mainly depends on productivity. Our results are consistent with our conceptual framework: in a context of contract incompleteness, the more important the specific inputs, the more likely the insourcing relative to outsourcing; moreover, the more productive the firm, the more appealing the foreign sourcing than domestic sourcing.

Our results are robust to alternative productivity and input specificity measures and various controls at the firm, industry, and province levels. Moreover, they survive in a wide range of econometric models—including probit, bivariate probit, and multivariate probit—and survey estimation methods.

Interestingly, our descriptive statistics reveal that the pandemic has adversely affected business for 89% of the sampled firms, irrespective of their geographical location, manufacturing activity, and size. These firms assign an important role to their supply chain in mitigating the impact of COVID-19. Moreover, the firm’s boundaries show a great deal of inertia; just a handful of firms plan to change their ownership and location decisions permanently because of the pandemic. Then, they plan to switch from insourcing to outsourcing and from foreign to domestic sourcing. This suggests that value chains will survive the COVID-19–induced permanent changes; however, we expect them to be less global in the future.

The rest of the paper is organised as follows. Section 2 introduces our conceptual framework. Section 3 provides background information about Lombardy. Section 4 describes data, empirical methodology, and results. Section 5 summarises our main findings, derives policy implications, and suggests future lines of research.

## Conceptual Framework

This section introduces the conceptual framework motivating our empirical analysis on the firm’s boundaries in times of COVID-19.

In our terminology, studying the firm’s boundaries means discussing which production tasks should be performed internally and externalised either in the domestic or in a foreign country. For simplicity, consider a stylised framework in which the production of a final good requires intermediate inputs. Accordingly, the final good producer takes two crucial decisions over sourcing. On one hand, it has to decide whether to manufacture the needed inputs by itself (make), committing to insourcing, or buy them from an independent input supplier (buy), committing to outsourcing. On the other hand, it has to decide whether to rely on domestic inputs (domestic), engaging in domestic sourcing, or depend on foreign inputs (foreign), engaging in foreign sourcing. We refer to the make-or-buy choice as the final good producer’s ownership decision and the domestic-or-foreign choice as its location decision. In this simple framework, studying the firm’s boundaries means addressing sourcing issues at the crossroad between ownership and location considerations.

The firm’s boundaries have been studied extensively in the last two decades from various perspectives, including international business, general management, supply chain or operations management, economic geography or sociology or regional studies, incomplete contracts theory, and international economics (Kano et al. 2020). Our conceptual framework is grounded on incomplete contracts theory and international economics studies.

**Fig. 1:**
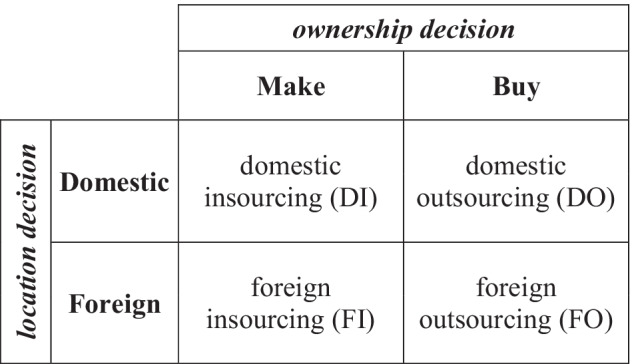
The boundaries of the firm as shaped by the ownership and location decisions

When globalisation was not relevant, sourcing was merely a local phenomenon, governed by ownership decisions alone. Final good producers decided whether to make or buy the needed inputs in the domestic country, committing to domestic insourcing (DI) in the former case and domestic outsourcing (DO) in the latter. DI and DO were the only instances of firms’ boundaries because the sourcing location was completely ignored (Fig. 1).

When merely a local phenomenon, sourcing could be completely understood through the incomplete contracts theories of insourcing. Formerly introduced by Coase (1937) and lately operationalised by Williamson (1985), the notions of transaction costs and contract incompleteness received the first formal treatment in Grossman and Hart (1986) and Hart and Moore (1990), starting the property rights theory of the firm. In an ideal world, the relationship between a final good producer and an input supplier would be governed by a complete contract. This contract specifies all the contingencies that may affect the contractual relationship. However, real-world contracts are incomplete because of unforeseen contingencies and the prohibitively high costs of contract writing and enforcing (Salaniè 1997). They are vague or silent on several key features and have gaps, missing provisions, or ambiguities (Grossman and Hart 1986). Contract incompleteness becomes of major concern when intermediate inputs require relation-specific investments, that is, prior investments that pay off more inside the final good producer-input supplier relationship than outside it. Relation-specific investments bind the input supplier and the final good producer, preventing them from switching freely to alternative partners in case of disagreement. The combination of contract incompleteness and relation-specific investments makes the input supplier underinvest because it fears to be held up, that is, to be denied the due payment by the final good producer claiming the occurrence of some contingencies uncovered by the contract. This undermines final good production because the producer can only manufacture a suboptimal amount of final good, relying on insufficient intermediate inputs. Anticipating this, the final good producer may decide to make the intermediate inputs to avoid hold-up concerns. However, engaging in DI entails higher production costs because the final good producer is less familiar with input manufacturing and thus less efficient than an independent input supplier. Therefore, considering its ownership decision, the final good producer trades off the benefits of maximal relation-specific investments (under DI) with the benefits of minimal production costs (under DO). A key prediction of the property rights theory of the firm, in this simple framework, is that relation specificity drives the final good producer’s ownership decision toward DI: the more relation-specific investment is needed to manufacture the intermediate inputs, the more likely is the DI option to secure against hold-up induced underinvestment. When globalisation is not an issue, a theory of insourcing settles the debate about the firm’s boundaries.

However, globalisation has become an issue nowadays. Therefore, sourcing can no longer be considered local. As a global phenomenon, sourcing is governed by the interplay between ownership and location decisions. Final good producers decide whether to make or buy the needed inputs, committing to insourcing in the former case and outsourcing in the latter. Moreover, final good producers decide whether to rely on domestic or foreign inputs, engaging in domestic sourcing in the former case and foreign sourcing in the latter. As summarised in Fig. 1, combining ownership and location considerations cause four instances of firms’ boundaries, DI, DO, foreign insourcing (FI), and foreign outsourcing (FO). As a global phenomenon, sourcing has been investigated in a few studies at the crossroad between incomplete contracts theory and international economics (for a survey, see Antras 2014; Gattai 2006; Spencer 2005). These contributions address the firm’s boundaries extending the property rights theory of the firm to the international context. Therefore, they expose the firm’s ‘black box’—traditionally explored by contract theorists—and simultaneously endogenise the market environment—as in the international economics tradition. From a theoretical perspective, the most important contributions are those by McLaren (2000), Grossman and Helpman (2002, 2003, 2005), Antras (2003), Ottaviano and Turrini (2007), and Antras and Helpman (2004). The framework, common to these theoretical models is that final good production requires relation-specific inputs that the firm procures under contract incompleteness. McLaren (2000) and Grossman and Helpman (2002) focused on the domestic side of the final good producer’s ownership decision (DI versus DO) in industry equilibrium models. Grossman and Helpman (2003), Antras (2003), and Ottaviano and Turrini (2007) analysed the foreign side of the final good producer’s ownership decision (FO versus FI). Grossman and Helpman (2005) studied the final good producer’s location decision (DO versus FO). Antras and Helpman (2004) addressed both ownership and location concerns in a joint theoretical framework. In their model, the ownership decision is sensitive to input specificity. In choosing between insourcing and outsourcing, final good producers trade off the benefits of maximal relation-specific investments under the former, with the benefits of minimal production costs under the latter. As relying on specific inputs is risky under contract incompleteness, firms employing specific inputs prefer insourcing.^1^ The location decision depends on productivity: in choosing between domestic sourcing and foreign sourcing, final good producers trade off the benefits of minimal fixed costs under the former, with minimal variable costs under the latter. As operating abroad is costlier than operating domestically, only the most productive firms can afford foreign sourcing costs. Assuming firms’ heterogeneity, à la Melitz (2003), Antras and Helpman (2004) showed that in low-tech sectors, insourcing never occurs: lower-productivity firms engage in DO, whereas higher-productivity firms engage in FO. However, in high-tech sectors, all sourcing strategies may be undertaken: lower-productivity firms rely on domestic inputs, and higher-productivity firms rely on foreign inputs; among firms that source in the same country, the most productive insource, and the least productive outsource.^2^

In the last decade, a burgeoning empirical literature has grown rapidly to test the main predictions of Antras and Helpman (2004). Depending on data availability, Tomiura (2007a), Defever and Toubal (2013), and Corcos et al. (2013) studied the relative attractiveness of FO and FI. Tomiura (2005, 2009) and Ito et al. (2011) analysed FO and DO. Tomiura (2007b), Federico (2010), Kohler and Smolka (2011, 2021), and Gattai and Trovato (2016) considered all sourcing strategies in a joint empirical framework. The available evidence confirms the main theoretical predictions of Antras and Helpman (2004): irrespective of the year and country of analysis, firms that commit to foreign sourcing are, on average, more productive than firms that commit to domestic sourcing. Moreover, insourcing firms are, on average, more productive than outsourcing firms.

To conclude, our conceptual framework delineated above suggests two testable predictions. Considering an incomplete contracts theory/international economics perspective on firm’s boundaries, Hypothesis 1 and Hypothesis 2 are summarised:

- **Hypothesis 1**: Relation-specific investments are major drivers of the final good producer’s ownership decision: the more specific the intermediate inputs, the more likely the make solution. Therefore, we expect firms relying more on specific inputs to engage in insourcing rather than in outsourcing.
- **Hypothesis 2**: Productivity is a major driver of the final good producer’s location decision: the more productive the firm, the more likely the foreign solution. Therefore, we expect more productive firms to engage in foreign sourcing rather than in domestic sourcing.

## Lombardy: Background Information

In this section, we provide some background information on Lombardy’s economy before the onset of the pandemic and briefly describe the impact of COVID-19 on regional business activities afterward.^3^

Located in the North of Italy, Lombardy is one of the most developed, industrialised, and open regions in Europe. Lombardy’s GDP per capita equalled 127% of the EU-27 average in 2019.^4^ Regarding industrialisation, in 2018, Lombardy counted 8582 active enterprises per 100,000 inhabitants, as compared with 5761 active enterprises per 100,000 inhabitants in the EU-27.

Trade openness is a distinctive feature of the Lombard economy. In 2019, Lombardy’s exports accounted for 27% of the Italian total and 36% of the regional value-added, well above the national share of 30%. The EU is the main destination market for Lombardy exporting firms, absorbing 66% of exports from the region. The USA and Asia follow, with shares in regional exports equal to 12% and 17%, respectively.^5^

Compared with European regions with similar economic and demographic characteristics, Lombardy performs better on exports’ extensive and intensive margin. Assolombarda (2019) presented evidence from a survey conducted in 2017 on a sample of 509 firms from Lombardy and four key productive European regions (Auvergne-Rhône-Alpes, Baden-Württemberg, Bayern, Cataluña). Lombardy’s share of exporting firms equals 77%, against 60% for the entire sample. Additionally, exports account for 41% of turnover for Lombardy exporting firms, above the 31% share of the entire sample.

Imports are equally important in explaining Lombardy’s degree of trade openness. In 2019, imports to Lombardy amount to 31% of the Italian total. The EU accounts for 74% of the region’s imports, followed by Asia with a 20% share.^6^ Survey data in Assolombarda (2019) suggest that the share of importing firms in Lombardy and the German regions is very similar (36–38%), albeit below the sample average (40%).

Participation in GVCs is one of the main drivers of Lombardy’s exports. Bentivogli et al. (2018) estimated that slightly over 50% of Lombardy’s gross exports and outflows towards other Italian regions originate from participation in GVCs. Furthermore, Lombardy’s share of foreign value-added from international sources is the highest among Italian regions, and bears witness to the importance of the region’s international backward linkages.

Survey data confirm the importance of global sourcing for Lombard firms, compared with firms from similar European regions. Assolombarda (2019) reported that 6.5% of Lombardy firms engage in foreign sourcing, a share consistent with firms from German regions and above the average among the sampled firms. Additionally, Lombard firms (6.4%) are more likely to engage in foreign outsourcing than sampled firms from other regions.

Lombardy was among the first European regions to register the presence of COVID-19. On February 21, 2020, the first COVID-19 patient in Italy was identified in the province of Lodi. On March 1, 2020, Lombardy counted 984 total cases. On April 1, 2020, the region reported 44,743 total cases. One year later (May 2021), Lombardy counted 8148 total cases per 100,000 inhabitants.^7^ The regional incidence was 20% above the national level but consistent with the data from France and Spain.^8^

The local government acted swiftly to stem the contagion. On March 7, 2020, Lombardy imposed severe restrictions on individual mobility and economic activities. The region went into lockdown: all non-essential commercial activities were ceased, and all non-essential production activities were suspended. Similar measures were enforced in the rest of the country in the following weeks. The strictest restrictions were lifted only on May 18, 2020, and commercial and production activities resumed gradually.^9^ However, the respite was short-lived. Contagions spiked again in the second half of October, and new and severe restrictions were introduced. Lastly, limitations on commercial activities were lifted in the region and most of the country on April 26, 2021.

Local and global economic conditions took a high toll on business activities in Lombardy. In 2020, the GDP in Lombardy was estimated to fall by 9.7% compared to 2019 (Assolombarda 2021a). The estimated fall for Lombardy was above the national one (− 8.9%) and exceeded the estimated fall for the EU-27 (− 6.1%). Data on industrial production show that the impact of the COVID-19 pandemic on the manufacturing sector in Lombardy was particularly severe. Compared to 2019, industrial production fell by 10% in the first quarter, 21% in the second quarter, and 5.2% in the third quarter of 2020. A 2.6% fall in the fourth quarter led to an estimated year-on-year fall slightly above 9% (Assolombarda 2021b).

In 2020, trade flows in and out of Lombardy suffered an equally important reduction due to the collapse of international trade. If the first quarter exports fell by 4.2%, the second quarter witnessed a fall by 27.3%. Exports remained below the 2019 level by 8% in the third quarter and 2.3% in the fourth quarter (Assolombarda 2021b). Year on year, exports from Lombardy fell by 10.6% in 2020. The fall in exports experienced by Lombard firms was above the national level (− 9.7%) but consistent with the fall registered in European regions with similar economic characteristics (Assolombarda 2021c).^10^

As for exports, in 2020, regional imports suffered an 11% fall compared to the previous year. The most severe fall (− 24.7%) was registered in the second quarter, and imports remained below the 2019 level in the following quarters.^11^

To conclude, Lombardy belongs to the industrial core of Europe, and its firms are very active in international markets. Lombardy was one of the first regions in Europe to be affected by the pandemic. Therefore, we consider an empirical investigation on the Lombard firms’ boundaries particularly informative on the prospects of GVCs in times and the aftermath of COVID-19.

## Empirical Analysis

### Data and Descriptive Statistics

The present study draws on an original survey, conducted between April and July 2020, of a representative sample of Italian manufacturing firms headquartered in Lombardy.

Our target sample of 300 firms is drawn from the last national firm census and stratified according to geographical location, manufacturing activity, and firm size. Geographical location stratification is based on four macro-areas that group neighbouring provinces according to their productive specialisation; they are designated as follows: Northwest (including Como, Lecco, and Varese), Northeast (including Bergamo, Brescia, and Sondrio), Southwest (including Lodi, Milano, Monza Brianza, and Pavia), and Southeast (including Cremona and Mantova). The manufacturing activity stratification follows the taxonomy of Pavitt (1984), grouping industries into four macro-categories according to the source of technology and technical change; they are designated as supplier-dominated, specialised-suppliers, science-based, and scale-intensive. Firm size stratification reflects the number of employees, and is based on three cells: firms with fewer than 10 employees, firms with 10–49 employees, and firms with more than 50 employees.

The number of firms in each stratum of the target sample was obtained to ensure proportionality to the total number of firms in the same stratum of the population.

All firms were contacted by phone, and a multiple-choice questionnaire was submitted by email to senior managers and CEOs.

The questionnaire consisted of two sections: in the first, we collect the background information of the firms; in the second, we investigate the firms’ sourcing strategies and reactions to the pandemic.

With a response rate of 70%, this study covers 212 enterprises. After omitting firms missing values of the relevant variables, our working sample consists of 203 firms. Table 1 shows that our working sample is highly representative of the entire population.

**Table 1:** Sample of respondents, working sample and population of Lombard enterprises, by geographical location, manufacturing activity, and firm size

	Class	Population		Sample of respondents		Working sample	
		*N*	%	*N*	%	*N*	%
Geographical location	Northwest	17,400	21	47	22	44	22
	Northeast	24,695	29	66	31	63	31
	Southwest	36,064	42	84	40	81	40
	Southeast	6553	8	15	7	15	7
	Total	84,712	100	212	100	203	100
Manufacturing activity	Supplier dominated	36,730	44	92	43	88	43
	Science based	9297	11	23	11	23	11
	Scale intensive	19,748	23	47	22	45	22
	Specialized suppliers	18,937	22	50	24	47	23
	Total	84,712	100	212	100	203	100
Firm size	0–9	65,630	77	164	77	156	77
	10–49	16,037	19	41	19	40	20
	≥ 50	3045	4	7	4	7	3
	Total	84,712	100	212	100	203	100

Source: elaborations on authors’ database

Regarding the geographical location, the majority of firms are from the Southwest area (40%), followed by the Northeast (31%), Northwest (22%), and Southeast (7%). This suggests that the manufacturing core of Lombardy is centred in Lodi, Milano, Monza Brianza, and Pavia, whereas Cremona and Mantova account for a limited share of the local business.

For the manufacturing activity, supplier-dominated operations are the main economic activity, involving 43% of the sampled firms. They are followed by the specialised suppliers (23%) and the scale intensive (22%) industries, whereas the science-based (11%) activities represent the smallest segment. These data confirm that the industrial texture of the region is highly diversified, with multiple specialisations leading to a balanced mixture of traditional and high-tech activities.

Finally, regarding firm size, our sample is characterised by the sharp prevalence of small enterprises (77%) with fewer than 10 employees. Medium and large firms account for a limited 20% and 3% of the total, respectively. Given the well-documented relevance of Lombardy for the Italian economy (ASR 2021), this suggests that a mass of small and medium enterprises, rather than a handful of huge conglomerates, is responsible for outstanding shares of the national value-added, GDP, export, and import.

We requested firms to report their core sourcing strategy in 2020, to portray their boundaries in times of COVID-19. Following Antras and Helpman (2004), we allowed firms’ boundaries to rise from a combination of ownership and location decisions.

**Fig. 2:**
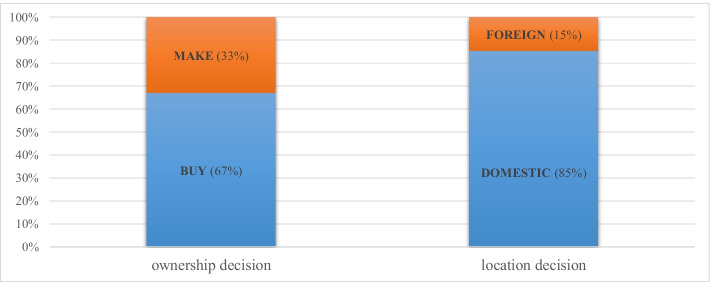
Ownership and location decisions of the sampled firms. Elaborations on authors’ database

**Fig. 3:**
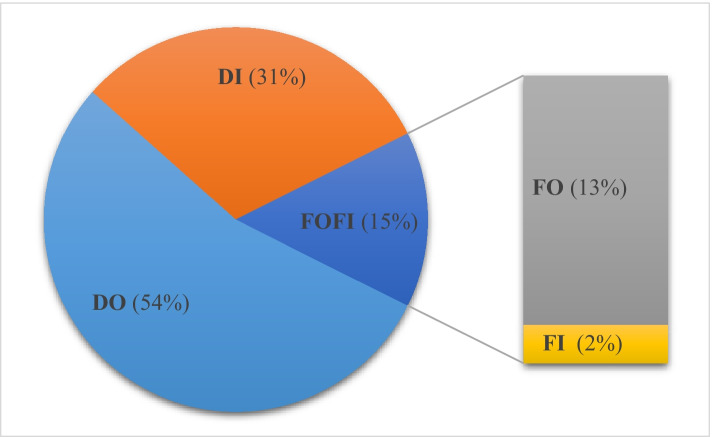
The boundaries of the sampled firms. Elaborations on authors’ database

Regarding ownership decision, Fig. 2 reveals that 67% of the sampled firms buy their inputs from independent suppliers, thus engaging in outsourcing (BUY), against 33% that manufacture the components themselves, preferring insourcing (MAKE). As for the location decision, 85% of our firms engage in domestic sourcing, employing ‘made in Italy’ components (DOMESTIC), whereas 15% prefer foreign sourcing, relying on foreign inputs (FOREIGN). If ownership and location decisions are combined, DO emerges as pervasive, accounting for 54% of the respondents; DI, FO, and FI follow with shares equal to 31%, 13%, and 2%, respectively. Given the small percentage of firms relying on FI, Fig. 3 groups the two instances of foreign sourcing under the same label, FOFI, covering both FI and FO. At this stage, it is worth mentioning that our results are robust to the stratification criteria, meaning that the strong preference for out- over insourcing and for domestic over foreign sourcing survives once we consider sub-samples by geographical location, manufacturing activity, and firm size. Moreover, these results are consistent with the empirical evidence of Assolombarda (2019).

For this research, our survey data have been complemented with balance sheet information downloaded from AIDA, a comprehensive database on Italian enterprises administered by Bureau van Dijk. This allows us to explain the firm’s boundaries through firm-level variables, testing Hypotheses 1 and 2.

According to Hypothesis 1, input specificity is a major driver of firms’ ownership decision. We expect firms relying more on specific inputs to engage in insourcing rather than outsourcing to secure against hold-up risks. *Input_specificity* is a dummy equal to 1 if firm *i* regards specific inputs as very relevant for its production process, drawing on our survey interviews.^12^ In the questionnaire, inputs are defined as ‘specific’ when tailored to a particular final good. Firms evaluated the relevance of specific inputs according to a 1–5 Likert scale, where 1 denotes minimal relevance and 5 denotes maximal relevance. Our dummy *input_specificity* is set equal to 1 when firm *i*’s evaluation is equal to 5.

According to Hypothesis 2, productivity is a major driver of firms’ location decision. We expect more productive firms to engage in foreign rather than domestic sourcing. Productivity, in its broadest interpretation, reflects the efficiency by which inputs are turned into outputs and can be defined as labour productivity or total factor productivity (Hulten 2001). Labour productivity—computed as the ratio between value-added and the number of employees—does not control for differences in capital intensity across firms, whereas total factor productivity does. Therefore, total factor productivity can describe the efficiency of resource use more accurately. Following Gal (2013), there are two empirical approaches to measure total factor productivity, the index number approach, and the estimation-based approach.^13^ This study evaluated labour productivity (*labour_productivity*) and total factor productivity (*TFP*) for robustness. Particularly, we measure *TFP* under an estimation-based approach applying the semi-parametric method of Levinsohn and Petrin (2003) to address the simultaneity and selection biases.^14^ Accordingly, we assume the production function of firm *i* at time *t* to be Cobb–Douglas. In this framework, the logarithm of firm *i*’s output at time *t* can be expressed as a function of the logarithm of the freely variable input labour, the logarithm of the intermediate input, and the logarithm of the state variable capital. Following Gal (2013), we measure the firm’s output in terms of value-added, the input labour as the number of employees, intermediate input as material costs, and capital stock as fixed assets. The entire 2015–2018 time series for value-added, number of employees, material costs, and fixed assets is exploited to apply the Levinsohn and Petrin (2003) approach, implementing the ‘levpet’ routine available in Stata. Nominal variables are deflated according to the most appropriate 2-digit industry-level deflators for capital, intermediate inputs, and value-added, available for Italy from the STAN-OECD (2020) database.

Table 2 displays the correlation matrix for our main variables of interest. *labour_productivity* and *TFP* are highly and positively correlated, meaning that they are alternative proxies of firm-level productivity and cannot be entered as regressors in the same equation. Conversely, *input_specificity* is correlated neither with *labour_productivity* nor with *TFP*. The correlation coefficient is indeed close to zero and displays a negative sign in both cases. This suggests that, in our sample, more productive firms do not systematically rely on specific inputs. We believe this is consistent with the industrial texture of Lombardy, where traditional supplier-dominated operations are the economic core and high-tech science-based activities represent the smallest segment. Although productivity is likely to vary across Pavitt sectors, with high-tech firms being more productive than traditional firms, this does not need to be the case for *input_specificity*. Firms regard specific inputs as very relevant when they employ fully tailored components. By definition, fully tailored components are designed for a particular final good; they can be sophisticated inputs delivered to high-tech firms or unsophisticated inputs delivered to traditional firms.^15^ Notably, *input_specificity* is equal to 1 (i.e., firms regard specific inputs as very relevant) for 40% of supplier-dominated firms, 44% of scale-intensive, 44% of specialized-suppliers, and 52% of science-based firms. This seems to suggest that *input_specificity* is relevant in our data, no matter the Pavitt industry. Put another way, this variable is a real firm-level proxy of relation-specific investments and does not capture industry-level patterns.

**Table 2:** Correlation matrix of core regressors

	*relation_specificity_conservative*	*relation_specificity_liberal*	*input_specificity*	*TFP*	*labor_productivity*
*relation_specificity_conservative*	1.0000				
*relation_specificity_liberal*	0.5015	1.0000			
*input_specificity*	0.0348	0.0390	1.0000		
*TFP*	-0.0805	0.0844	-0.0428	1.0000	
*labor_productivity*	-0.1528	0.0231	-0.0655	0.7852	1.0000

Source: elaborations on authors’ database

Given that *input_specificity* is almost uncorrelated with either *labour_productivity* or *TFP*, it can be entered as regressor in the same equation including our measure of firm-level productivity.

Table 3 compares the input specificity and productivity of the sampled firms by ownership and location decisions. For every variable, Table 3 displays the number of observations and the mean in the groups of firms that engage in insourcing (MAKE) versus outsourcing (BUY) and in foreign sourcing (FOREIGN) versus domestic sourcing (DOMESTIC). Mean comparison tests reveal whether differences in the means are positive or negative and statistically significant or insignificant.

**Table 3:** Productivity and input specificity differentials of the sampled firms by ownership and location decisions

Variable	Working sample		By ownership decision					By location decision				
			MAKE		BUY		MAKE-BUY	FOREIGN		DOMESTIC		FOREIGN-DOMESTIC
	Obs	Mean	Obs	Mean	Obs	Mean	*t* test	Obs	Mean	Obs	Mean	*t* test
*input_specificity*	203	0.44	67	0.54	136	0.39	0.15**	30	0.33	173	0.46	− 0.13
*labour_productivity*	194	73.75	60	73.39	134	73.91	− 0.52	28	106.72	166	68.19	38.53***
*TFP*	184	1.37	57	1.3	127	1.4	− 0.10	27	1.74	157	1.31	0.43**

*, **, and *** mean significant at 10%, 5%, and 1% levels, respectively

Source: elaborations on authors’ database

A preliminary investigation of the data suggests that insourcing is associated with higher input specificity than outsourcing. This suggests that MAKE firms systematically differ from BUY firms in terms of *input_specificity*. Consistent with Hypothesis 1, the former exhibit higher mean values of *input_specificity* than the latter, and differences in the means (MAKE-BUY) are positive and statistically significant. Moreover, Table 3 shows that foreign sourcing is associated with higher productivity than domestic sourcing. FOREIGN firms systematically differ from DOMESTIC firms in productivity, irrespective of our measure. Consistent with Hypothesis 2, *labour_productivity* and *TFP* exhibit higher mean values in the former group. Our mean comparison tests reveal that differences in the means (FOREIGN-DOMESTIC) are positive and statistically significant.

Evidence is consistent when considering the firm’s boundaries, thus addressing ownership and location decisions in a joint empirical framework. Consistent with Fig. 3, Table 4 provides the number of observations and the means of input specificity and productivity in the groups of firms engaging in DO, DI, and FOFI. We consider DO as the baseline category, thus investigating systematic differences in *labour_productivity*, *TFP*, and *input_specificity* of DI and FOFI firms compared to DO firms. Consistent with Hypothesis 1, firms engaging in DI rely on specific inputs more than firms engaging in DO, and *input_specificity* differentials (DI-DO) are positive and statistically significant. Consistent with Hypothesis 2, firms engaging in FOFI exhibit higher productivity than firms engaging in DO, and productivity differentials (FOFI-DO) are positive and statistically significant.

**Table 4:** Productivity and input specificity differentials of the sampled firms by firms’ boundaries

Variable	Working sample		By BOUNDARIES							
			DO		DI		DI-DO	FOFI		FOFI-DO
	Obs	Mean	Obs	Mean	Obs	Mean	*t* test	Obs	Mean	*t* test
*input_specificity*	203	0.44	110	0.41	63	0.54	0.13*	30	0.33	− 0.08
*labour_productivity*	194	73.75	109	65.34	57	73.63	8.29	28	106.72	41.38***
*TFP*	184	1.37	104	1.3	53	1.33	0.03	27	1.74	0.44*

*, **, and *** mean significant at 10%, 5%, and 1% levels, respectively

Source: elaborations on authors’ database

### Contract incompleteness and the boundaries of the firm in times of COVID-19

#### Baseline models

In this sub-section, we explore the role of productivity and input specificity in shaping firms’ boundaries in times of COVID-19 more formally through econometric analysis.

As extensively described in Section 3, firms’ boundaries result from the interplay between the ownership (make-or-buy) and location (domestic-or-foreign) decisions governing sourcing. Therefore, Eqs. (1) and (2) are set as follows:

1 $${MAKE}_{it}=\alpha {{input\_specificity}_{it-2}}+\beta {productivity}_{it-2}+\gamma {firm\_ctrl}_{it}+\delta {province\_ctrl}_{it}+\theta {industry\_ctrl}_{it}+{\epsilon }_{it}$$

2 $${FOREIGN}_{it}=\alpha {{input\_specificity}_{it-2}}+\beta {productivity}_{it-2}+\gamma {firm\_ctrl}_{it}+\delta {province\_ctrl}_{it}+\theta {industry\_ctrl}_{it}+{\epsilon }_{it}$$

In Eq. (1), the dependent variable *MAKE* captures firm *i*’s ownership decision. Particularly, *MAKE* is a dummy variable: it equals 1 if firm *i* manufactures the inputs by itself, meaning that it engages in insourcing; it equals 0 if firm *i* buys the inputs from an independent supplier, meaning that it engages in outsourcing.

In Eq. (2), the dependent variable *FOREIGN* captures firm *i*’s location decision. *FOREIGN* is a dummy variable: it equals 1 if firm *i* employs foreign inputs, meaning that it engages in foreign sourcing; it equals 0 if firm *i* employs domestic inputs, meaning that it engages in domestic sourcing.

On the right-hand side of Eqs. (1) and (2), *input_specificity* and *productivity* denote our core regressors, consistent with the conceptual framework delineated in Section 3 and the definitions provided in sub-Section 4.1. According to Hypothesis 1, we expect *input_specificity* to influence firm *i*’s ownership decision: *input_specificity* should be a positive and statistically significant determinant of *MAKE* in Eq. (1). According to Hypothesis 2, we expect productivity to affect firm *i*’s location decision: *labour_productivity* and *TFP* should be positive and statistically significant determinants of *FOREIGN* in Eq. (2).

Additional controls at the firm-, province-, and industry-level are considered in Eqs. (1) and (2) to check the robustness of our results. Firm-level controls, grouped in the vector *firm_ctrl* include *age*, *group*, *white_collars*, and *size*. The variable *age* is defined as the difference between 2020 and firm *i*’s year of foundation; *group* is a dummy equal to 1 if firm *i* belongs to a business group, and 0 otherwise; *white_collars* captures the number of white collars and *size* the total number of employees.

Province-level and industry-level controls are accounted for with the geographical location and manufacturing activities dummies employed for stratification.^16^

All dependent variables refer to 2020, whereas the core regressors date back to 2018 to avoid simultaneity. Unfortunately, the cross-sectional design of our data does not allow us to implement rigorous econometric methods, apart from lagged variables, to account for endogeneity. Therefore, one should not interpret regressions as indicating the exact direction of causality but as a convenient way of summarising the statistical regularities among variables.

One concern with our data is a potentially high degree of multicollinearity among regressors. To make sure our estimates do not suffer from the multicollinearity trap, Table 5 reports the results of proper multicollinearity tests. The Variance Inflation Factor (VIF) and Tolerance (1/VIF) values are displayed for our core regressors in the estimated specifications. Since VIF (Tolerance) values are lower (larger) than 10 (0.1), we conclude that multicollinearity is not an issue with our data.

**Table 5:** Multicollinearity tests

*input_specificity*	1.01	1.00	1.06	1.04
	(0.9858)	(0.9989)	(0.9432)	(0.9589)
*labor_productivity*	1.01		1.13	
	(0.9858)		(0.8846)	
*TFP*		1.00		1.55
		(0.9989)		(0.6456)
*firm_ctrl*			1.15	1.20
			(0.8704)	(0.8343)
*industry_ctrl*			1.57	1.60
			(0.6393)	(0.6271)
*province_ctrl*			1.56	1.57
			(0.6540)	(0.6489)
Overall	1.01	1.00	1.33	1.39
	0.9901	1.00	0.7519	0.7194

VIF and Tolerance (in parenthesis) values are displayed for regressors. Mean VIF and mean Tolerance (in parenthesis) are displayed for firm, industry and country controls and the overall set of regressors

Source: elaborations on authors’ database

Our results from probit estimates of Eqs. (1) and (2) are shown in Table 6.^17^ In column (a), we consider parsimonious specifications in which *MAKE* and *FOREIGN* are regressed only on *input_specificity* and *productivity*; in column (b), we introduce richer specifications in which firm-, province- and industry-level controls are included. Coefficients, marginal effects (in square brackets), and *p* values (in round brackets) are displayed for the main variables of interest.

**Table 6:** Probit estimates of Eqs. (1) and (2)

	*MAKE*				*FOREIGN*			
	*(a)*		*(b)*		*(a)*		*(b)*	
*input_specificity*	0.4447	0.4051	0.6172	0.5310	− 0.1638	− 0.2459	− 0.2055	− 0.1863
	[0.1568]	[0.1434]	[0.2088]	[0.1802]	[− 0.0359]	[− 0.0542]	[− 0.0392]	[− 0.0358]
	(0.020)**	(0.038)**	(0.003)***	(0.012)**	(0.480)	(0.300)	(0.427)	(0.477)
*labour_productivity*	0.0003		0.0003		0.0032		0.0031	
	[0.0001]		[0.0001]		[0.0007]		[0.0006]	
	(0.802)		(0.872)		(0.040)**		(0.070)*	
*TFP*		− 0.0606		− 0.2617		0.1840		0.3063
		[− 0.0212]		[− 0.0878]		[0.0413]		[0.0597]
		(0.559)		(0.123)		(0.081)*		(0.054)*
*firm_ctrl*	No	No	Yes	Yes	No	No	Yes	Yes
*industry_ctrl*	No	No	Yes	Yes	No	No	Yes	Yes
*province_ctrl*	No	No	Yes	Yes	No	No	Yes	Yes
Obs	194	184	191	181	194	184	191	181
*R*^2^	0.023	0.021	0.099	0.090	0.037	0.029	0.120	0.127

Coefficients, marginal effects (in square brackets), and *p* values (in round brackets) are displayed. *, **, and *** mean significant at 10%, 5%, and 1% levels, respectively

Source: elaborations on authors’ database

*Input_specificity* turns out to be positive and statistically significant in every specification of Eq. (1). This implies that the more the firm relies on specific inputs, the more likely the insourcing is than outsourcing, consistent with Antras and Helpman (2004). *input_specificity* is a key determinant of our firms’ ownership decision, confirming Hypothesis 1. In light of predictions of Antras and Helpman (2004), the lack of significance of productivity in Eq. (1) may appear puzzling. However, productivity plays a role in the ownership decision only if the fixed costs of vertical integration and outsourcing differ (Helpman 2011, ch. 6). In particular, more productive firms opt for insourcing only if the fixed costs of vertical integration exceed those of outsourcing, as in Antras and Helpman (2004). Since the ordering of fixed costs of vertical integration and outsourcing is ultimately an empirical matter, our results are consistent with the theoretical framework of Antras and Helpman (2004).^18^

*productivity* is positive and statistically significant in every specification of Eq. (2), suggesting that higher productivity firms are more likely to engage in foreign rather than domestic sourcing, consistent with the theoretical framework of Antras and Helpman (2004). *productivity* is a key driver of our firms’ location decision, confirming Hypothesis 2. Notably, these results are robust to alternative productivity measures—*labour_productivity* versus *TFP*—and specifications—parsimonious versus rich—thus offering a clear-cut overview of firms’ boundaries.^19^ Regarding control variables, they exhibit a low predictive power in Eqs. (1) and (2), except *age* (*white_collars*) that is negatively (positively) and significantly correlated with *MAKE*. This evidence suggests that younger firms are more prone to insourcing than outsourcing; the same is true for firms employing several white collars.

The firm’s ownership and location decisions might be related to some extent. In our data, this is evident from the fact that the intersection between *MAKE* and *FOREIGN* is not empty.^20^ To account for this possibility, we estimate a bivariate probit model with the following system of equations:

$${MAKE}_{it}=\alpha {{input\_specificity}_{it-2}}+\beta {productivity}_{it-2}+\gamma {firm\_ctrl}_{it}+\delta {province\_ctrl}_{it}+\theta {industry\_ctrl}_{it}+{\epsilon }_{it}$$

3 $${FOREIGN}_{it}=\alpha \alpha {{input\_specificity}_{it-2}}+\beta {productivity}_{it-2}+\gamma {firm\_ctrl}_{it}+\delta {province\_ctrl}_{it}+\theta {industry\_ctrl}_{it}+{\epsilon }_{it}$$

All the dependent and independent variables are as in Eqs. (1) and (2). Moreover, we retain the same specifications—parsimonious (column a) versus rich (column b)—to facilitate comparisons with previous results.

Estimating Eq. (3) in a bivariate probit setting, we allow regressors to jointly influence the probability that firm *i* engages in *MAKE* and *FOREIGN*. Consequently, for every specification, Table 7 displays two columns: the first one describes the impact of covariates on *MAKE*, whereas the second captures their effects on *FOREIGN*. Coefficients, marginal effects (in square brackets), and *p* values (in round brackets) are displayed for the main variables of interest, together with the correlation coefficient *rho* and the *p* value for the likelihood test of *rho* equal to 0. Considering *rho* in a bivariate probit setting might be informative. In fact, *rho* is the correlation coefficient between the residuals of the two equations. It indicates the correlation between unobserved factors affecting *MAKE* and *FOREIGN*. Therefore, considering this parameter is crucial to identify the most appropriate estimation method. If *rho* is statistically different from 0, then a bivariate probit is better than a pair of univariate probits. Table 7 reveals that *rho* is significantly different from 0 in every specification, meaning that the bivariate probit is the most appropriate model for our data.

**Table 7:** Bivariate probit estimates of Eq. (3)

	*MAKE*	*FOREIGN*	*MAKE*	*FOREIGN*	*MAKE*	*FOREIGN*	*MAKE*	*FOREIGN*
	*(a)*		*(a)*		*(b)*		*(b)*	
*input_specificity*	0.4500	− 0.1538	0.4062	− 0.2455	0.6468	− 0.1394	0.5373	− 0.1694
	[0.0078]	[0.0078]	[0.0037]	[0.0037]	[0.0091]	[0.0091]	[0.0090]	[0.0090]
	(0.019)**	(0.505)	(0.038)**	(0.299)	(0.002)***	(0.587)	(0.010)**	(0.515)
*labour_productivity*	0.0003	0.0033			0.0001	0.0034		
	[0.0001]	[0.0001]			[0.0001]	[0.0001]		
	(0.835)	(0.045)**			(0.970)	(0.060)*		
*TFP*			− 0.0674	0.1771			− 0.2749	0.2830
			[0.0053]	[0.0053]			[0.0018]	[0.0018]
			(0.525)	(0.094)*			(0.107)	(0.066)*
*firm_ctrl*	No		No		Yes		Yes	
*industry_ctrl*	No		No		Yes		Yes	
*province_ctrl*	No		No		Yes		Yes	
Obs	194		184		191		181	
Wald Chi^2^	10.07		7.99		35.53		31.52	
*rho*	− 0.4165		− 0.2891		− 0.5676		− 0.3501	
*p* value *rho*	0.008***		0.066*		0.002***		0.048**	

Coefficients, marginal effects (in square brackets), and *p* values (in round brackets) are displayed. *, **, and *** mean significant at 10%, 5%, and 1% levels, respectively

Source: elaborations on authors’ database

Notably, results from Table 7 are consistent with the evidence reported in Table 6, in that *input_specificity* is a key driver of firm *i*’s ownership decision, whereas *productivity* affects firm *i*’s location decision.

These results are robust to firm-, province- and industry-level controls, and they hold irrespective of the productivity measure, supporting Hypothesis 1 and Hypothesis 2.

Further, in our identification strategy, we estimate Eq. (4):

4 $${BOUNDARIES}_{it}=\alpha {{input\_specificity}_{it-2}}+\beta {productivity}_{it-2}+\gamma {firm\_ctrl}_{it}+\delta {province\_ctrl}_{it}+\theta {industry\_ctrl}_{it}+{\epsilon }_{it}$$

In Eq. (4), the dependent variable *BOUNDARIES* is a discrete variable that captures the specific instance of firm *i*’s boundaries at the interplay between ownership and location decisions. Consistent with Antras and Helpman (2004), *BOUNDARIES* is equal to 0 if firm *i* engages in DO; it is equal to 1 if firm *i* engages in DI; it is equal to 2 if firm *i* engages in FOFI. Data limitations prevent us from splitting foreign insourcing and foreign outsourcing into separate categories of the discrete variable *BOUNDARIES*. As mentioned in sub-Section 4.1, only 2% of our firms engage in foreign insourcing; therefore, for robustness, we club the two instances of FOFI into the same category *BOUNDARIES* = 2.

Given the discrete nature of the dependent variable *BOUNDARIES*, regressions are conducted in a multivariate probit framework, setting DO as the baseline category.^21^ To allow for comparisons with our previous evidence, on the right-hand side of Eq. (4), we consider the same set of covariates already exploited in Eqs. (1), (2), and (3). Moreover, parsimonious versus rich specifications are accounted for in columns (a) and (b), respectively.

Results from our multivariate probit estimates of Eq. (4) are summarised in Table 8. Coefficients, marginal effects (in square brackets), and *p* values (in round brackets) are displayed for the main variables of interest.

**Table 8:** Multivariate probit estimates of Eq. (4)

	*BOUNDARIES*							
	*(a)*				*(b)*			
	*DI*	*FOFI*	*DI*	*FOFI*	*DI*	*FOFI*	*DI*	*FOFI*
*input_specificity*	0.5496	0.0037	0.4954	− 0.1242	0.7363	0.0039	0.6534	− 0.0133
	[0.1401]	[− 0.0355]	[0.1336]	[− 0.0536]	[0.1687]	[− 0.0399]	[0.1492]	[− 0.0367]
	(0.044)**	(0.991)	(0.076)*	(0.704)	(0.013)**	(0.991)	(0.030)**	(0.971)
*labour_productivity*	0.0034	0.0059			0.0034	0.0059		
	[0.0005]	[0.0008]			[0.0005]	[0.0007]		
	(0.196)	(0.029)**			(0.272)	(0.058)*		
*TFP*			0.0502	0.2537			− 0.1942	0.3625
			[− 0.0037]	[0.0407]			[− 0.0634]	[0.0658]
			(0.740)	(0.090)*			(0.418)	(0.095)*
*firm_ctrl*	No		No		Yes		Yes	
*industry_ctrl*	No		No		Yes		Yes	
*province_ctrl*	No		No		Yes		Yes	
Obs	194		184		191		181	
Wald Chi^2^	9.61		7.45		33.33		30.84	

Coefficients, marginal effects (in square brackets), and *p* values (in round brackets) are displayed. *, **, and *** mean significant at 10%, 5%, and 1% levels, respectively

Source: elaborations on authors’ database

Consistent with our previous evidence, the probability of firm *i* engaging in DI rather than DO is crucially governed by *input_specificity*, consistent with Hypothesis 1. Concurrently, the probability of firm *i* engaging in FOFI, rather than DO, is driven by *productivity*, consistent with Hypothesis 2. This evidence is robust to firm-, province- and industry-level controls and holds irrespective of the productivity measure.

Jointly considered, our findings suggest that the firm’s boundaries are shaped by the interplay of *input_specificity* and *productivity*: an increase in the former induces domestically oriented firms to insource to mitigate hold-up concerns; an increase in the latter enables firms to cross national borders, thus engaging in FOFI.

#### Robustness checks

In this sub-section, we consider a few of robustness checks, to explore the sensitivity of our results to survey estimation methods and alternative measures of input specificity.^22^

As for the first check, we re-estimate Eqs. (1), (2), (3), and (4) using survey estimation methods to control for the potential bias originating from the response rate. Each combination of a single geographical location (out of four) and a single manufacturing activity (out of four) denotes a stratum (16 in total). In the econometric analysis, we use sampling information to obtain consistent and efficient estimates and draw conclusions about Lombardy as a whole. Following Cusmano et al. (2010), Kohler and Smolka (2011), and Gattai and Trovato (2016), we weigh each observation by the inverse of the probability of being sampled using, for every stratum, location-, and industry-specific information on the total number of firms in the population and the sample.

Our results are consistent with those presented previously, in line with Hypothesis 1 and Hypothesis 2. Particularly, our probit estimates of Eqs. (1) and (2), displayed in Table 9, are consistent with those shown in Table 6. Our bivariate probit estimates of Eq. (3), displayed in Table 10, are consistent with those reported in Table 7. Our multivariate probit estimates of Eq. (4), displayed in Table 11, are aligned with those provided in Table 8, confirming that the firm’s boundaries in times of COVID-19 are driven by *input_specificity* and *productivity*, the former affecting the sampled firms’ ownership decision, the latter governing their location decision.

**Table 9:** Weighted probit estimates of Eqs. (1) and (2)

	*MAKE*				*FOREIGN*			
	*(a)*		*(b)*		*(a)*		*(b)*	
*input_specificity*	0.4273	0.4006	0.5752	0.4985	− 0.1619	− 0.2428	− 0.1974	− 0.1662
	[0.1517]	[0.1427]	[0.1959]	[0.1704]	[− 0.0348]	[− 0.0526]	[− 0.0360]	[− 0.0303]
	(0.029)**	(0.044)**	(0.006)***	(0.019)**	(0.488)	(0.311)	(0.386)	(0.482)
*labour_productivity*	0.0003		− 0.00001		0.0036		0.0035	
	[0.0001]		[− 0.0001]		[0.0008]		[0.0006]	
	(0.998)		(0.967)		(0.017)**		(0.079)*	
*TFP*		− 0.0593		− 0.2423		.1908		0.3012
		[− 0.0210]		[− 0.0823]		[.0419		[0.0555]
		(0.532)		(0.137)		(0.051)*		(0.025)**
*firm_ctrl*	No	No	Yes	Yes	No	No	Yes	Yes
*industry_ctrl*	No	No	Yes	Yes	No	No	Yes	Yes
*province_ctrl*	No	No	Yes	Yes	No	No	Yes	Yes
Obs	194	184	191	181	194	184	191	181
*R*^2^	0.021	0.020	0.102	0.091	0.047	0.032	0.141	0.144

Coefficients, marginal effects (in square brackets), and *p* values (in round brackets) are displayed. *, **, and *** mean significant at 10%, 5%, and 1% levels, respectively

Source: elaborations on authors’ database

**Table 10:** Weighted bivariate probit estimates of Eq. (3)

	*MAKE*	*FOREIGN*	*MAKE*	*FOREIGN*	*MAKE*	*FOREIGN*	*MAKE*	*FOREIGN*
	*(a)*		*(a)*		*(b)*		*(b)*	
*input_specificity*	0.4314	− 0.1526	0.4012	− 0.2423	0.6049	− 0.1333	0.5051	− 0.1503
	[0.0076]	[0.0076]	[0.0036]	[0.0036]	[0.0083]	[0.0083]	[0.0085]	[0.0085]
	(0.027)**	(0.512)	(0.044)**	(0.312)	(0.003)***	(0.550)	(0.017)**	(0.525)
*labour_productivity*	− 0.0001	0.0038			− 0.0003	0.0038		
	[0.0001]	[0.0001]			[0.0001]	[0.0001]		
	(0.971)	(0.020)**			(0.876)	(0.069)*		
*TFP*			− 0.0649	0.1844			− 0.2529	0.2778
			[0.0059]	[0.0059]			[0.0020]	[0.0020]
			(0.510)	(0.060)*			(0.124)	(0.026)**
*firm_ctrl*	No		No		Yes		Yes	
*industry_ctrl*	No		No		Yes		Yes	
*province_ctrl*	No		No		Yes		Yes	
Obs	194		184		191		181	
Wald Chi^2^	14.81		9.79		48.45		43.27	
*rho*	− 0.3914		− 0.2738		− 0.5513		− 0.3467	
*p* value *rho*	0.020**		0.093*		0.001***		0.048**	

Coefficients, marginal effects (in square brackets), and *p* values (in round brackets) are displayed. *, **, and *** mean significant at 10%, 5%, and 1% levels, respectively

Source: elaborations on authors’ database

**Table 11:** Weighted multivariate probit estimates of Eq. (4)

	*BOUNDARIES*							
	*(a)*				*(b)*			
	*DI*	*FOFI*	*DI*	*FOFI*	*DI*	*FOFI*	*DI*	*FOFI*
*input_specificity*	0.5351	0.0026	0.4977	− 0.1178	0.6802	− 0.0041	0.6089	− 0.0046
	[0.1375]	[− 0.0342]	[0.1348]	[− 0.0520]	[0.1567]	[− 0.0360]	[0.1397]	[− 0.0315]
	(0.055)*	(0.994)	(0.080)*	(0.722)	(0.021)**	(0.990)	(0.044)**	(0.989)
*labour_productivity*	0.0032	0.0064			0.0031	0.0063		
	[0.0004]	[0.0008]			[0.0004]	[0.0007]		
	(0.202)	(0.007)***			(0.278)	(0.025)**		
*TFP*			0.0517572	0.2640			− 0.1807	0.3630
			[− 0.0037]	[0.0415]			[− 0.0597]	[0.0623]
			(0.714)	(0.061)*			(0.434)	(0.049)**
*firm_ctrl*	No		No		Yes		Yes	
*industry_ctrl*	No		No		Yes		Yes	
*province_ctrl*	No		No		Yes		Yes	
Obs	194		184		191		181	
Wald Chi^2^	11.85		8.21		42.61		41.96	

Coefficients, marginal effects (in square brackets), and *p* values (in round brackets) are displayed. *, **, and *** mean significant at 10%, 5%, and 1% levels, respectively

Source: elaborations on authors’ database

As for the second check, we acknowledge that our *input_specificity* variable relies on firms’ self-reported measure of relation specificity. Ideally, one would employ firm-level objective measures of input specificity, including capital intensity, skill intensity and technological intensity of the inputs employed by the firm (Jabbour and Kneller 2010; Jabbour 2012). Alternatively, input specificity could be proxied by the number of competitors in the market for the firm’s final product, the rationale being that the fewer the firms, the likelier the occurrence of hold-up problems (González-Díaz et al. 2000; Mazzanti et al. 2009, 2011). However, firm-level objective measures of input specificity are very demanding in terms of data and thus subject to misreporting by firms. This suggests relying on industry-level objective measures of input specificity.

Drawing on previous literature, there are several approaches to measure relation specificity at the industry level. For example, one could consider the complexity of production in a certain industry, as proxied by capital intensity or skill requirements (Costinot et al. 2011; Costinot 2009). Alternatively, relation specificity could be measured in terms of contract intensity, that is, by the prevailing mode of exchange of the inputs employed in a certain industry (Nunn 2007). Our data allow us to rely on the latter approach, adopting the taxonomy of industries developed by Nunn (2007) along the above lines.

In Nunn (2007), the importance of relation specificity is quantified by defining a variable that measures, for each good, the proportion of its inputs that requires relation-specific investments. This results in a two-step process: first, using input–output tables, we determine which inputs are used and in what proportion in the production of each final good; second, inputs requiring relation-specific investments are identified using Rauch (1999) classification of inputs sold on organized exchange, reference priced or neither. If an input is sold on an organized exchange, the market for this input is thick, with many buyers and sellers; therefore, the scope for hold-up is limited and relation specificity is low. If an input is reference priced in trade publications, there exists a reasonably large number of potential buyers and sellers^23^; hold-up might be an issue with intermediate level of relation specificity. If an input is neither sold on an organized exchange nor reference priced, the scope for hold-up is wide and relation specificity is high.^24^

Following Nunn (2007), we introduce two measures of industry-level relation specificity, denoted as *relation_specificity_conservative* and *relation_specificity_liberal*. The former minimizes the proportion of inputs considered as relation specific by focusing on inputs that are neither sold on an organized exchange nor reference priced. The latter maximizes instead the proportion of inputs considered as relation specific by accounting for those not sold on an organized exchange.^25^ Our measures of industry-level relation specificity are computed as follows:

5 $${relation\_specificity\_conservative}_{j}=\sum_{y}{\theta }_{jy}{R}_{y}^{neither}$$

6 $${relation\_specificity\_liberal}_{j}=\sum_{y}{\theta }_{jy}{(R}_{y}^{neither}+ {R}_{y}^{reference\; priced})$$

In Eqs. (5) and (6), *j* denotes the 1-digit industry, and *y* the single input used in industry *j*. ${\theta }_{jy}$ captures the relative importance of input *y* in industry *j*; it is defined as the ratio of the value of input *y* used in industry *j* over the value of all inputs used in industry *j*, according to the input–output tables. ${R}_{y}^{neither}$(${R}_{y}^{reference\; priced}$) denotes the proportion of input *y* that is relation specific according to the Rauch (1999)’s classification of inputs that are neither sold on an organized exchange nor reference priced (reference priced). In light of the above discussion, an increase in *relation_specificity_conservative* or *relation_specificity_liberal* should be interpreted as an increase in relation specificity.

At this stage, it is worth mentioning that these measures are defined at the 1-digit level. Therefore, they can be entered in econometric specifications including industry controls by means of manufacturing activities dummies as in sub-Section 4.2.1. Notice also that *relation_specificity_conservative* and *relation_specificity_liberal* are correlated with each other but not correlated with *input_specificity*, *labour_productivity* and *TFP*. Therefore, we can add a measure of industry-level relation specificity to the econometric specifications considered in sub-Section 4.2.1.

Our results are consistent with those presented previously, confirming the relevance of Hypothesis 1 and Hypothesis 2. Particularly, our probit estimates of Eqs. (1) and (2), shown in Table 12, point to the importance of *input_specificity* in orienting the ownership decision and productivity in shaping the location decision, as previously observed in Table 6. However, controlling for *relation_specificity_conservative*/*relation_specificity_liberal* provides some additional remarks. As for ownership, our industry measures of relation specificity turn out to be insignificant, whereas our firm measure of input specificity remains significant consistently through the different specifications.^26^ This seems to suggest that the ownership decision is a matter of firm-, more than industry-level determinants. As for location, controlling for *relation_specificity_conservative*/*relation_specificity_liberal* does not undermine significance of either *labour_productivity* or *TFP*. However, our industry measures of relation specificity tend to play a negative role in orienting the location decision, being negatively significant in most specifications. This seems to suggest that firms belonging to more relation-specific industries tend to engage in domestic rather than foreign sourcing, possibly to contain the hold-up risk.

**Table 12:** Probit estimates of Eqs. (1) and (2), measures of industry-level relation specificity included

	*MAKE*							
	*(a)*				*(b)*			
*input_specificity*	0.829	0.4483	0.4124	0.4117	0.6205	0.6173	0.5310	0.5313
	[0.1565]	[0.1579]	[0.1458]	[0.1456]	[0.2096]	[0.2088]	[0.1801]	[0.1803]
	(0.021)**	(0.020)**	(0.036)**	(0.036)**	(0.003)***	(0.003)***	(0.012)**	(0.012)**
*relation_specificity_conservative*	− 0.3464		− 0.5176		0.4193		0.0164	
	[− 0.1213]		[− 0.1814]		[0.1407]		[0.0055]	
	(0.527)		(0.352)		(0.606)		(0.984)	
*relation_specificity_liberal*		− 0.7989]		− 0.7760		.0245		− 0.3176
		[− 0.2797]		[− 0.2720]		[.0082]		[− 0.1066]
		(0.235)		(0.361)		(0.982)		(0.779)
*labor_productivity*	0.0003	0.0005			.0003	.0003		
	[0.0001]	[0.0002]			[.0001]	[.0001]		
	(0.829)	(0.735)			(0.858)	(0.873)		
*TFP*			− 0.0646	− 0.0516			− 0.2617	− 0.2637
			[− 0.0226]	[− 0.0181]			[− 0.0878]	[− 0.0885]
			(0.530)	(0.619)			(0.123)	(0.121)
*firm_ctrl*	No	No	No	No	Yes	Yes	Yes	Yes
*industry_ctrl*	No	No	No	No	Yes	Yes	Yes	Yes
*province_ctrl*	No	No	No	No	Yes	Yes	Yes	Yes
Obs	194	194	184	184	191	191	181	181
*R*^2^	0.024	0.027	0.025	0.025	0.100	0.099	0.090	0.090

	*BUY*							
	*(a)*				*(b)*			
*input_specificity*	− 0.1611	− 0.1324	− 0.2283	− 0.2133	− 0.1803	− 0.2038	− 0.1511	− 0.1723
	[− 0.0344]	[− 0.0278]	[− 0.0493]	[− 0.0446]	[− 0.0333]	[− 0.0373]	[− 0.0285]	[− 0.0320]
	(0.492)	(0.576)	(0.340)	(0.381)	(0.493)	(0.441)	(0.570)	(0.520)
*relation_specificity_conservative*	− 1.3058		− 1.1513		− 1.9393		− 1.5596	
	[− 0.2817]		[− 0.2531]		[− 0.3618]		[− 0.2974]	
	(0.052)*		(0.086)*		(0.052)*		(0.119)	
*relation_specificity_liberal*		− 2.4968		− 2.7509		− 2.9166		− 2.8791
		[− 0.5282]		[-.5841]		[− 0.5402]		[− 0.5411]
		(0.005)***		(0.003)***		(0.017)**		(0.028)**
*labor_productivity*	0.0031	0.0037			0.0029	0.0033		
	[− 0.2835]	[0.0008]			[0.0005]	[0.0006]		
	(0.059)*	(0.026)**			(0.113)	(0.077)*		
*TFP*			0.1684	0.2172			0.2737	0.2746
			[0.0370]	[0.0461]			[0.0522]	[0.0516]
			(0.123)	(0.048)**			(0.097)*	(0.087)*
*firm_ctrl*	No	No	No	No	Yes	Yes	Yes	Yes
*industry_ctrl*	No	No	No	No	Yes	Yes	Yes	Yes
*province_ctrl*	No	No	No	No	Yes	Yes	Yes	Yes
Obs	194	194	184	184	191	191	181	181
*R*^2^	0.061	0.084	0.049	0.086	0.146	0.158	0.144	0.160

Coefficients, marginal effects (in square brackets), and *p* values (in round brackets) are displayed. *, **, and *** mean significant at 10%, 5%, and 1% levels, respectively

Source: elaborations on authors’ database

Our bivariate probit estimates of Eq. (3), displayed in Table 13, are consistent with those reported in Table 7. *input_specificy* is confirmed as the main driver of the ownership decision and *productivity* plays a positive role in promoting foreign sourcing, in line with Hypothesis 1 and Hypothesis 2. *relation_specificity_conservative* and *relation_specificity_liberal* do not seem to matter for the make-or-buy choice; however, they matter for domestic-foreign choice, pushing towards domestic sourcing.

**Table 13:** Bivariate probit estimates of Eq. (3), measures of industry-level relation specificity included

	*MAKE*	*FOREIGN*	*MAKE*	*FOREIGN*	*MAKE*	*FOREIGN*	*MAKE*	*FOREIGN*
	*(a)*		*(a)*		*(a)*		*(a)*	
*input_specificity*	0.4489	− 0.1457	0.4472	− 0.1124	0.4111	− 0.2277	0.4089	− 0.2077
	[0.0082]	[0.0082]	[0.0099]	[0.0099]	[0.0049]	[0.0049]	[0.0062]	[0.0062]
	(0.019)**	(0.532)	(0.020)**	(0.633)	(0.036)**	(0.340)	(0.037)**	(0.392)
*relation_specificity_conservative*	− 0.3650	− 1.3307			− 0.5050	− 1.1363		
	[− 0.0525]	[− 0.0525]			[− 0.0673]	[− 0.0673]		
	(0.507)	(0.049)**			(0.365)	(0.092)*		
*relation_specificity_liberal*			− 0.7374	− 2.4429			− 0.7225	− 2.6691
			[− 0.1042]	[− 0.1042]			[− 0.1438]	[− 0.1438]
			(0.368)	(0.006)***			(0.399)	(0.004)***
*labor_productivity*	0.0002	0.0032	0.0004	0.0038				
	[0.0001]	[0.0001]	[0.0001]	[0.0001]				
	(0.865)	(0.071)*	(0.776)	(0.032)**				
*TFP*					− 0.0707	− 0.5050	− 0.0579	0.2059
					[0.0043]	[0.0043]	[0.0068]	[0.0068]
					(0.501)	(0.152)	(0.585)	(0.061)*
*firm_ctrl*	No		No		No		No	
*industry_ctrl*	No		No		No		No	
*province_ctrl*	No		No		No		No	
Obs	194		194		184		184	
Wald Chi^2^	14.10		18.82		11.82		17.40	
*rho*	− 0.4491		− 0.4399		− 0.3056		− 0.2978	
*p* value* rho*	0.005***		0.005***		0.054*		0.059*	

	*MAKE*	*FOREIGN*	*MAKE*	*FOREIGN*	*MAKE*	*FOREIGN*	*MAKE*	*FOREIGN*
	*(b)*		*(b)*		*(b)*		*(b)*	
*input_specificity*	0.6539	− 0.0942	0.6433	− 0.1328	0.5376	− 0.1191	0.5378	− 0.1470
	[0.0102]	[0.0102]	[0.0102]	[0.0102]	[0.0106]	[0.0106]	[0.0102]	[0.0102]
	(0.002)***	(0.720)	(0.002)***	(0.613)	(0.010)***	(0.654)	(0.010)***	(0.580)
*relation_specificity_conservative*	0.4922	− 2.0381			0.0737	− 1.6994		
	[− 0.0328]	[− 0.0328]			[− 0.0524]	[− 0.0524]		
	(0.549)	(0.042)**			(0.931)	(0.095)*		
*relation_specificity_liberal*			0.1462	− 2.6083			− 0.2550	− 2.7512
			[− 0.0570]	[− 0.0570]			[− 0.0994]	[− 0.0994]
			(0.890)	(0.032)**			(0.822)	(0.036)**
*labor_productivity*	0.0002	0.0032	0.0001	0.0035				
	[0.0001]	[0.0001]	[0.0001]	[0.0001]				
	(0.908)	(0.105)	(0.930)	(0.075)*				
*TFP*					− 0.2706	0.2423	− 0.2744	0.2474
					[0.0005]	[0.0005]	[0.0006]	[0.0006]
					(0.111)	(0.127)	(0.107)	(0.109)
*firm_ctrl*	Yes		Yes		Yes		Yes	
*industry_ctrl*	Yes		Yes		Yes		Yes	
*province_ctrl*	Yes		Yes		Yes		Yes	
Obs	191		191		181		181	
Wald Chi^2^	37.72		38.98		33.51		35.65	
*rho*	− 0.5790		− 0.5420		− 0.3712		− 0.3433	
*p* value *rho*	0.002***		0.003***		0.037**		0.055*	

Coefficients, marginal effects (in square brackets), and *p* values (in round brackets) are displayed. *, **, and *** mean significant at 10%, 5%, and 1% levels, respectively

Source: elaborations on authors’ database

Lastly, our multivariate probit estimates of Eq. (4) are displayed in Table 14. Evidence is fully consistent with our previous results, testifying to the importance of *input_specificity, labour_productivity*, *TFP*, *relation_specificity_conservative*, and *relation_specificity_liberal* in assessing the firm’s boundaries in times of COVID-19.

**Table 14:** Multivariate probit estimates of Eq. (4), measures of industry-level relation specificity included

	*BOUNDARIES*							
	*(a)*							
	*DI*	*FOFI*	*DI*	*FOFI*	*DI*	*FOFI*	*DI*	*FOFI*
*input_specificity*	0.5497	0.0112	0.5764	0.07950	0.5089	− 0.0925	0.5254	− 0.0501
	[0.1392]	[− 0.0341]	[0.1406]	[− 0.0255]	[0.1345]	[− 0.0489]	[0.1353]	[− 0.0425]
	(0.045)**	(0.973)	(0.037)**	(0.810)	(0.070)*	(0.780)	(0.063)*	(0.882)
*relation_specificity_conservative*	− 0.7308	− 1.9716			− 1.0606	− 1.8939		
	[− 0.0571]	[− 0.2779]			[− 0.1442]	[− 0.2527]		
	(0.354)	(0.035)**			(0.185)	(0.042)**		
*relation_specificity_liberal*			− 2.4695	− 4.3693			− 2.6473	− 4.7290
			[− 0.3356]	[− 0.5356]			[− 0.3562]	[− 0.5958]
			(0.042)**	(0.001)***			(0.037)**	(0.000)***
*labor_productivity*	0.0034	0.0058	0.0043	0.0072				
	[0.0005]	[0.0007]	[0.0006]	[0.0009]				
	(0.200)	(0.037)**	(0.125)	(0.015)**				
*TFP*					0.0451	0.2320	0.0874	0.3148
					[− 0.0037]	[0.0365]	[0.0014]	[0.0455]
					(0.768)	(0.137)	(0.573)	(0.047)**
*firm_ctrl*	No		No		No		No	
*industry_ctrl*	No		No		No		No	
*province_ctrl*	No		No		No		No	
Obs	194		194		184		184	
Wald Chi^2^	13.49		19.69		11.66		19.25	

	*BOUNDARIES*							
	*(b)*							
	*DI*	*FOFI*	*DI*	*FOFI*	*DI*	*FOFI*	*DI*	*FOFI*
*input_specificity*	0.7420	0.0382	0.7485	0.02310	0.6585	0.0359	0.6691	0.0212
	[0.1674]	[− 0.0342]	[0.1695]	[− 0.0370]	[0.1476]	[− 0.0293]	[0.1502]	[− 0.0320]
	(0.012)**	(0.916)	(0.012)**	(0.950)	(0.029)**	(0.922)	(0.027)**	(0.954)
*relation_specificity_conservative*	0.0546	− 2.4523			− 0.3674	− 2.1997		
	[0.1440]	[− 0.36346]			[0.0326]	[− 0.3109]		
	(0.963)	(0.079)*			(0.763)	(0.113)		
*relation_specificity_liberal*			− 1.5552	− 4.5746			− 1.9685	− 4.7428
			[− 0.1081]	[− 0.5737]			[− 0.1960]	[− 0.5887]
			(0.318)	(0.010)**			(0.239)	(0.012)**
*labor_productivity*	0.0030	0.0055	0.0032	0.0060				
	[0.0004]	[0.0006]	[0.0004]	[0.0007]				
	(0.331)	(0.084)*	(0.295)	(0.056)*				
*TFP*					− 0.2134	0.3164	− 0.2183	0.3113
					[− 0.0651]	[0.0588]	[− 0.0657]	[0.0569]
					(0.377)	(0.158)	(0.365)	(0.154)
*firm_ctrl*	Yes		Yes		Yes		Yes	
*industry_ctrl*	Yes		Yes		Yes		Yes	
*province_ctrl*	Yes		Yes		Yes		Yes	
Obs	191		191		181		181	
Wald Chi^2^	35.62		38.86		32.80		36.48	

Coefficients, marginal effects (in square brackets), and *p* values (in round brackets) are displayed. *, **, and *** mean significant at 10%, 5%, and 1% levels, respectively

Source: elaborations on authors’ database

#### Discussion

In sub-Section 4.2.2, we analysed the firm’s boundaries in times of COVID-19. We studied the role of input specificity and productivity in shaping our firms’ solutions to sourcing presently. However, the pandemic produces medium- and long-run effects on GVCs, making it challenging to study the firm’s boundaries in the aftermath of COVID-19, that is, to ask what will be of our firms’ solutions to sourcing in the future.^27^

Our data are not suitable to address this issue through rigorous econometric analysis. However, they allow providing some descriptive evidence to portray the potential evolution of sourcing in the aftermath of the pandemic.

Interviews reveal that COVID-19 has affected 89% of the sample firms, irrespective of their geographical location, manufacturing activity, and size. Additionally, over 50% of firms in our sample report having suffered supply disruptions during the pandemic.

**Fig. 4:**
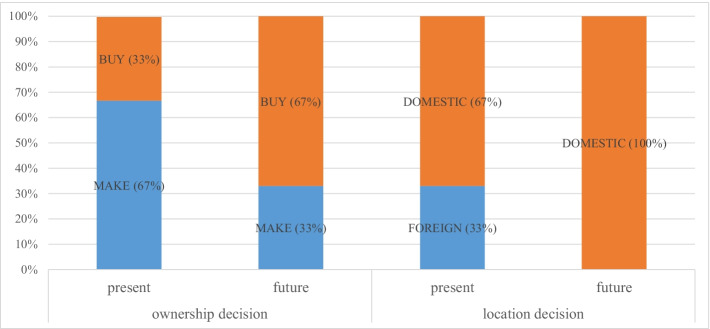
Ownership and location decisions of the COVID-19–induced permanent switchers at present and in the future. Elaborations on authors’ database

However, the firm’s boundaries show a great deal of inertia because just a handful of firms (11) are rethinking their solutions to sourcing. Moreover, out of 11 future switchers, only three plan to change their ownership and location decisions permanently. We refer to these firms as the ‘COVID-19-induced permanent switchers’ and compare their ownership and location decisions presently and in the future (Fig. 4).

All sourcing strategies are likely to be permanently rethought because of COVID-19. Considering firms’ ownership decision, we appreciate a slight decrease in insourcing in favour of outsourcing. Regarding firms’ location decisions, our switchers plan to abandon foreign sourcing completely and engage in domestic sourcing alone.

Our findings highlight that Lombard enterprises do not fear relying on independent suppliers, albeit they fear relying on distant suppliers in the future.

**Fig. 5:**
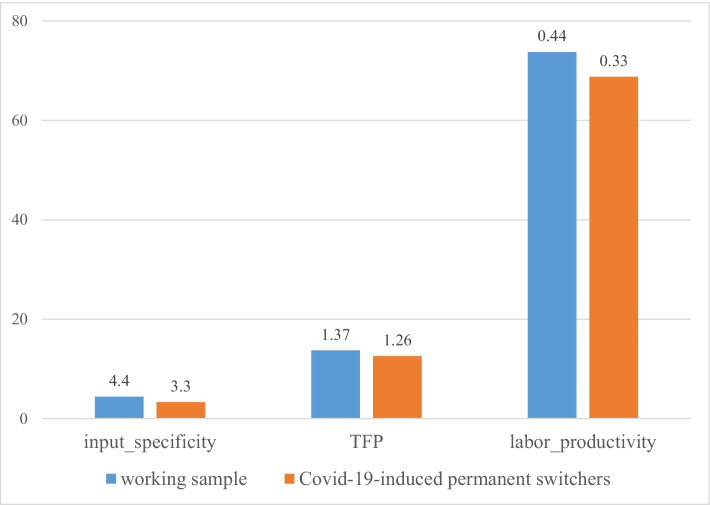
Productivity and input specificity of the COVID-19–induced permanent switchers versus the overall working sample. Source: elaborations on authors’ database

Lack of data prevents us from analysing the firm’s boundaries in the aftermath of COVID-19 through econometric regressions. However, we believe that some insights might emerge from investigating the characteristics of the COVID-19–induced permanent switchers, in light of Hypotheses 1 and 2. According to Hypothesis 1, input specificity is a major driver of firms’ ownership decision; according to Hypothesis 2, productivity is a major driver of firms’ location decision. Our previous descriptive statistics (4.1) and econometric analysis (4.2.1, 4.2.2) confirm a positive and statistically significant correlation between *input_specificity* and engagement in insourcing on one hand and between productivity and engagement in foreign sourcing, on the other hand. From Fig. 5, the COVID-19–induced permanent switchers are below the overall working sample mean of *input_specificity* and productivity—measured as *labour_productivity* and *TFP*. Therefore, their plan to switch from insourcing to outsourcing and to abandon foreign sourcing and engage in domestic sourcing alone is consistent with Hypotheses 1 and 2.

Following Giovannetti et al. (2011), the persistence of ownership and location decisions should not come as a surprise. Firms invest large amounts of relational capital in forging supply chains, and such investments pay off in times of crisis, evident from our data.

**Fig. 6:**
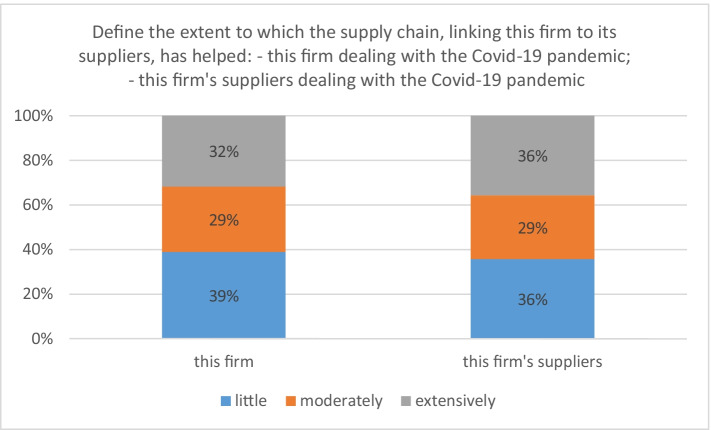
Importance of the supply chain in mitigating the adverse consequences of COVID-19. Source: elaborations on authors’ database

In survey interviews, Lombard firms define the extent to which their supply chain helped them and their suppliers deal with the COVID-19 pandemic. Notably, the supply chain was helpful for the majority of respondents. Considering the firm’s (the firm’s suppliers’) perspective, 32% (36%) of respondents believe that the supply chain helped extensively in dealing with the pandemic, followed by 29% (29%) declaring that it helped moderately and 39% (36%) reporting little help (Fig. 6).^28^ Our evidence is fully consistent when dissected by the stratification criteria; this suggests that our respondents credit the supply chain as majorly important during the pandemic, irrespective of their geographical location, manufacturing activity, and firm size.

## Conclusions

In this study, we adopt an incomplete contracts theory/international economics perspective to study the firm’s boundaries in times of COVID-19. We present new empirical evidence from survey interviews, conducted between April and July 2020, of a sample of 212 Italian manufacturing firms headquartered in Lombardy, one of the most developed regions in Europe and one of the first severely affected by the pandemic. Stratified by size, manufacturing activity, and province and with a response rate of 70%, our sample provides robust evidence on the firm’s boundaries at present and in the future.

Descriptive statistics and econometric analysis reveal that domestic outsourcing is the prevalent sourcing strategy among firms, followed by domestic integration, foreign outsourcing, and foreign integration. Considering the incomplete contracts theory/international economics perspective, we explain the preference for outsourcing over insourcing in terms of input specificity and domestic over foreign sourcing in terms of productivity. Our evidence confirms that input specificity is a positive and statistically significant determinant of insourcing, driving firms’ ownership decisions. In contrast, productivity is a positive and statistically significant determinant of foreign sourcing, shaping firms’ location decisions. Consistent with the theoretical model of Antras and Helpman (2004), our results are robust to alternative measures of productivity and various controls at the firm, industry, and province level. Moreover, they survive in a wide range of econometric models—including probit, bivariate probit, and multivariate probit—survey estimation methods and industry-level measures of relation specificity.

Our evidence reveals the pervasiveness of the adverse effects of the pandemic: 89% of firms in our sample report a negative impact of the pandemic on their business. No relevant differences emerge when considering geographical location, manufacturing activity, and size. While firms in our sample report that they incurred supply disruptions in the pandemic, their boundaries are characterised by a great deal of inertia. Just a handful of firms plan to change their ownership and location decisions permanently because of the pandemic. Then, they expect to switch from insourcing to outsourcing and from foreign to domestic sourcing. Consistent with our conceptual framework, the COVID-19–induced permanent switchers are less dependent on specific inputs and less productive than the sample average. Therefore, they do not fear relying on independent suppliers, albeit they fear relying on distant suppliers in times of crisis.

We believe that the above results are particularly informative in assessing the response of firms to the present and future challenges; the COVID-19 pandemic poses threats to their boundaries and ultimately to GVCs. Irrespective of their sourcing strategy, firms invest large amounts of relational capital in forging supply chains. Our evidence suggests that such investments pay off in times of crises, as our firms report that their supply chain played an important role in mitigating the impact of COVID-19. This might explain why firms resist changes in their ownership and location decisions in the aftermath of COVID-19.

Our results have implications for the policy debate on GVCs, examining whether excessive globalisation has created new economic vulnerabilities. While the world is changing because of the pandemic, some experts call for the end of GVCs, urging firms to re-engineer their boundaries in response to the crises (Javorcik 2020). Others argue that GVCs will continue to dominate the post-COVID-19 arena (Kowalski 2020) and alert that shorter supply chains would not be less vulnerable (Miroudot 2020). Our results suggest that value chains will survive the COVID-19 crisis, evident from the fact that the vast majority of our firms do not plan to modify their sourcing strategies.

However, we expect value chains to be less global after the crisis. This is evident from the fact that firms planning to modify their sourcing strategies will shift from foreign to domestic sourcing, relying exclusively on domestic inputs in the future.

To conclude, we acknowledge some limitations of our current analysis that restrict its scope. First, there is an issue of external validity. Although our sample is highly representative of the Lombard population of firms, it focuses on a single region within a single country. To better assess the robustness of our results, it would be preferable to widen the sample used for empirical purposes, possibly relying on cross-country comparisons. Second, our data have a cross-sectional nature, which prevents a proper causal analysis of the impact of COVID-19 on the firm’s boundaries. Rigorously addressing endogeneity issues would require enlarging the sample used for empirical purposes, possibly relying on time-series comparisons. Future research might improve on these limitations.

## Data Availability

The work elaborates survey interview data and Orbis data. Survey interview data were obtained under a confidentiality agreement and cannot be shared with third party. Orbis data are covered by a proprietary agreement between the Bureau van Dijk and Università degli Studi di Milano-Bicocca.

## Footnotes

1 Specific inputs are those tailored to a particular final good. They cannot be easily replaced if unavailable.

2 Antras and Helpman (2008) allow for different degrees of contract incompleteness. They show that improvements in contractibility of inputs either can encourage or discourage FO relative to FI.

3 Since the first cases of COVID-19 were reported in February 2020, we focus on Lombardy's economic performance in 2019.

4 If not otherwise stated, our data are the most recently available from Eurostat Homepage.

5 See Unioncamere Lombardia Homepage.

6 See Unioncamere Lombardia Homepage.

7 See Osservatorio nazionale della salute nelle regioni italiane Homepage.

8 We refrain from further cross-country comparisons because of differences in the COVID-19 data collection procedures.

9 The fall in electricity consumption is a fair indicator of the extent and severity of production disruptions caused by the pandemic in the first half of 2020. Year on year, weekly electricity consumption in Lombardy fell by 16% in March and 21% in April. In the months May–July, it was down by 13% on average. See Assolombarda (2021b).

10 Year on year, in 2020, exports fell by 10.3% in Cataluña, 10.6% in Auvergne-Rhône-Alpes, and 11.3% in Bayern. Baden-Württemberg experienced a milder reduction (− 7.3%).

11 In the third quarter, imports experienced a 10.7% reduction with respect to 2019, whereas the reduction in the fourth quarter was − 2.8%. For more information, see Unioncamere Homepage.

12 Following Masten et al. (1989, 1991) and Lyons (1995), we rely on firm’s self-reported measures of input specificity. Nonetheless, we include in our estimates an industry-level measure of input specificity to account for self-reporting biases. See Section 4.2.2.

13 The former is less demanding in terms of data requirement but more demanding in terms of theoretical assumptions, because it requires the production function to exhibit constant returns to scale; the opposite holds true for the latter.

14 For a discussion on the benefits of the Levinsohn and Petrin method, the reader is referred to Petrin et al. (2004).

15 Consider, for example, the fashion industry, which is a typical “made in Italy” business. In this case, firms rely on specific inputs, because they employ certain yarns, fabrics, buttons, etc. Still, they are very traditional firms, belonging to a textbook supplier-dominated industry.

16 See sub-Section 4.1.

17 Results are fully consistent when estimating Eqs. (1) and (2) with a logit rather than a probit model. More results are available from the authors upon request.

18 Defever and Toubal (2013) report survey data on French MNCs, confirming higher fixed cost of outsourcing vs. insourcing.

19 Still, it is worth mentioning that the lack of significance of our firm-level measure of input specificity attenuates when we adopt an industry-level measure. See sub-Section 4.2.2.

20 See sub-Section 4.1.

21 Results are fully consistent when estimating Eq. (4) with a multivariate logit rather than a multivariate probit model. More results are available from the authors upon request.

22 We thank an anonymous reviewer for this suggestion.

23 Trade publications are only released if there is a sufficiently high number of purchasers of the publication.

24 For an alternative measure of contract-intensity at the industry level, see Bernard et al. (2010).

25 This group encompasses inputs that have a reference price in trade publications and inputs that are neither sold on an organized exchange nor reference priced.

26 We tried also different specifications in which *relation_specificity_conservative* or *relation_specificity_liberal* replaced *input_specificity* rather than being added. However, those measures of industry-level relation specificity turned out to be insignificant in the ownership decision. More results are available from the authors upon request.

27 See Antras (2020) and Javorcik (2020) on this issue.

28 The extent to which supply chain helped with the COVID-19 pandemic is defined with respect to a 1–5 Likert scale, with 1 = minimal extent and 5 = maximal extent. In the following figures, ‘extensively’ means an evaluation equal to 4 or 5; ‘moderately’ an evaluation equal to 3 and ‘little’ an evaluation equal to 1 or 2.

## Code Availability

Code is available from the authors upon request.
